# *RUN1* and *REN1* Pyramiding in Grapevine (*Vitis vinifera* cv. Crimson Seedless) Displays an Improved Defense Response Leading to Enhanced Resistance to Powdery Mildew (*Erysiphe necator*)

**DOI:** 10.3389/fpls.2017.00758

**Published:** 2017-05-12

**Authors:** Mario Agurto, Rudolf O. Schlechter, Grace Armijo, Esteban Solano, Carolina Serrano, Rodrigo A. Contreras, Gustavo E. Zúñiga, Patricio Arce-Johnson

**Affiliations:** ^1^Laboratorio de Biología Molecular y Biotecnología Vegetal, Departamento de Genética Molecular y Microbiología, Facultad de Ciencias Biológicas, Pontificia Universidad Católica de ChileSantiago, Chile; ^2^Laboratorio de Fisiología y Biotecnología Vegetal, Departamento de Biología, Facultad de Química y Biología y CEDENNA, Universidad de Santiago de ChileSantiago, Chile

**Keywords:** *Vitis vinifera*, *Erysiphe necator*, *REN1*, *RUN1*, resistance, pyramiding, plant breeding, molecular marker-assisted selection

## Abstract

Fungal pathogens are the cause of the most common diseases in grapevine and among them powdery mildew represents a major focus for disease management. Different strategies for introgression of resistance in grapevine are currently undertaken in breeding programs. For example, introgression of several resistance genes (R) from different sources for making it more durable and also strengthening the plant defense response. Taking this into account, we cross-pollinated P09-105/34, a grapevine plant carrying both *RUN1* and *REN1* pyramided loci of resistance to *Erysiphe necator* inherited from a pseudo-backcrossing scheme with *Muscadinia rotundifolia* and *Vitis vinifera* ‘Dzhandzhal Kara,’ respectively, with the susceptible commercial table grape cv. ‘Crimson Seedless.’ We developed *RUN1REN1* resistant genotypes through conventional breeding and identified them by marker assisted selection. The characterization of defense response showed a highly effective defense mechanism against powdery mildew in these plants. Our results reveal that *RUN1REN1* grapevine plants display a robust defense response against *E. necator*, leading to unsuccessful fungal establishment with low penetration rate and poor hypha development. This resistance mechanism includes reactive oxygen species production, callose accumulation, programmed cell death induction and mainly *VvSTS36* and *VvPEN1* gene activation. *RUN1REN1* plants have a great potential as new table grape cultivars with durable complete resistance to *E. necator*, and are valuable germplasm to be included in grape breeding programs to continue pyramiding with other sources of resistance to grapevine diseases.

## Introduction

Grapevine (*Vitis vinifera* L.) is one of the most important fruit crops worldwide. It is affected by a large number of pathogenic microorganisms causing severe diseases with detrimental effects on yield and grape quality (Armijo et al., 2016b). The most common and important diseases affecting grapevine are caused by fungi and, among them, powdery mildew represents a major focus for disease management efforts in all the wine and table grape producing regions. Its etiologic agent corresponds to the biotrophic fungus *Erysiphe necator* Schw. [syn. *Uncinula necator* (Schw.) Burr.] (Gadoury et al., 2012). This pathogen can infect all green tissues of plant, showing a white-grayish powder as an easily recognizable symptom on the surface of infected leaves, stems, buds, flowers, and young fruits (Bendek et al., 2002; Calonnec et al., 2004). Its infection strategy starts with conidia germination on plant tissue surfaces to form a germ tube and lobed appressorium followed by the development of a penetration peg and subsequent invasion. Effective penetration continues with the development of feeding structures or haustoria, by which the fungus retrieves nutrients and secretes effectors that suppress host defenses, allowing the colonization of plant tissue surfaces by the development of secondary hypha. Finally it produces dissemination structures or conidiophores, which then sporulate to infect other host tissues and start a new infection cycle (Campbell et al., 2003; Glawe, 2008; Dry et al., 2010; Gadoury et al., 2012; Qiu et al., 2015).

*Erysiphe necator* corresponds to an obligate pathogen of the Vitaceae family and is the only powdery mildew species adapted to *V. vinifera*. Practically all *V. vinifera* cultivars are highly susceptible to powdery mildew, nevertheless, several Vitaceae species have developed resistance mechanisms against this fungus but lack commercial qualities (Riaz et al., 2007; Glawe, 2008; Dry et al., 2010; Gadoury et al., 2012). In this context, resistant genotypes become a valuable germplasm to be included in grapevine breeding programs. These natural powdery mildew resistance sources correspond to some North American and Asian genotypes, and the resistance trait is related to their evolutionary history, as described by several works (Riaz et al., 2007; Hoffmann et al., 2008; Coleman et al., 2009; Dry et al., 2010; Feechan et al., 2011; Ramming et al., 2011; Blanc et al., 2012; Gadoury et al., 2012; Qiu et al., 2015; Pap et al., 2016).

Knowledge of the resistance traits at the genetic level is essential to reach a significant improvement through plant breeding strategies. Several grapevine powdery mildew resistance loci have been identified and described to date. In this context, the dominant locus *RUN1* (Resistance to *Uncinula necator* 1) from *Muscadinia rotundifolia* has been successfully introgressed into *V. vinifera* plants. It was mapped to a region in chromosome 12 and also co-segregates with a grapevine downy mildew resistance locus named *RPV1* (Resistance to *Plasmopara viticola* 1) (Barker et al., 2005; Molnár et al., 2007; Dry et al., 2010; Gadoury et al., 2012). Feechan et al. (2013a) identified the genes responsible for these resistances, *MrRUN1* and *MrRPV1*, coding for TIR-NB-LRR proteins and becoming the first cloned and functionally characterized resistance genes in grapevine. *MrRUN1*-mediated defense response is associated with the induction of programmed cell death (PCD) at penetrated epidermal cells in a range of 24–48 hours post-inoculation (hpi) (Dry et al., 2010; Feechan et al., 2013a). On the other hand, the dominant locus *REN1* (Resistance to *Erysiphe necator* 1) belongs to ‘Kishmish Vatkana’ and ‘Dzhandzhal Kara,’ two Central Asian *V. vinifera* cultivars. It was mapped to linkage group 13, but the gene responsible for conferring resistance against the powdery mildew has not been identified to date. The enrichment of NBS-LRR and Cinnamyl Alcohol Dehydrogenase (CAD) genes in the region near the closest SRR marker has been described. *REN1*-mediated resistance mechanism involves the restriction of hyphal development, decreased conidiophore production and delayed PCD at the infection site (Hoffmann et al., 2008; Coleman et al., 2009). To date, other described resistance loci to grapevine powdery mildew are *RUN2* (Riaz et al., 2011; Feechan et al., 2015), *REN2* (Dalbó et al., 2001; Feechan et al., 2015), *REN3* (Welter et al., 2007), *REN4* (Ramming et al., 2011), *REN5* (Blanc et al., 2012), *REN6* and *REN7* (Pap et al., 2016).

Incompatible grapevine-*E. necator* interaction would be orchestrated by effector-triggered immunity (ETI) response, that restricts the development of the phytopathogenic fungus (Qiu et al., 2015). Hypersensitive response (HR) plays a crucial role in this type of plant defense, along with the occurrence of a strong oxidative burst, accumulation of callose and lignin deposits, increment of salicylic and jasmonic acid concentration (SA and JA, respectively), production of antimicrobial compounds, induction of PCD at the site of infection and also expression of pathogenesis-related proteins (*PR*s) in order to limit the fungus nutrient uptake and finally inhibit pathogen dispersal (Thatcher et al., 2005; Hammond-Kosack and Jones, 2015; Qiu et al., 2015). However, molecular mechanisms underlying plant immune response against grapevine powdery mildew are not fully understood.

We hypothesize that grapevine plants carrying the pyramided *RUN1* and *REN1* loci improve defense response against *E. necator*, resulting in increased restriction of fungal development in comparison with one single locus resistant genotypes.

With the aim to elucidate the grapevine defense response mediated by the pyramiding of *RUN1* and *REN1* resistance loci and achieve advances in the development of potential new *V. vinifera* cultivars with effective and durable resistance to *E. necator*, we cross-pollinated P09-105/34, a grapevine plant carrying both resistance loci inherited from a pseudo-backcrossing scheme with *M. rotundifolia* and *V. vinifera* ‘Dzhandzhal Kara,’ with the susceptible commercial table grape cv. ‘Crimson Seedless’ and obtained resistant genotypes to characterize the mechanisms involved in the defense response against powdery mildew.

## Materials and Methods

### Plant and Fungal Material

To generate grapevine plants containing two resistant loci against *E. necator*, *RUN1* (Barker et al., 2005) and/or *REN1* (Hoffmann et al., 2008), we crossed P09-105/34 and *V. vinifera* ‘Crimson Seedless.’ P09-105/34 is a progeny plant from the cross 91-4/27 × 02-2/81 obtained in collaboration with the Research Institute of Viticulture and Enology (University of Pécs, Hungary), where 91-4/27 corresponds to a segregating plant from *V. vinifera* ‘Dzhandzhal Kara’ × *V. vinifera* ‘Laszta,’ and 02-2/81 from the sixth pseudo-backcross of *M. rotundifolia* × *V. vinifera* (**Supplementary Figure S1**).

Plants used in this study correspond to nine selected offsprings grown in the greenhouses of the Laboratory of Plant Molecular Biology and Biotechnology, Department of Molecular Genetics and Microbiology, College of Biological Sciences, Pontifical Catholic University of Chile, Santiago.

In order to obtain replicates for the experiments, all the vines used in this study were propagated by herbaceous cuttings dipped in 100 mg per liter of indole-3-butyric acid (IBA) for 15 min to induce rooting, planted in pots containing peat and vermiculite mixture and maintained in greenhouse conditions with a 16/8 h photoperiod and 24 ± 2°C. To ensure availability of suitable leaves, plants were constantly pruned.

Fungal material used in this study corresponded to isolates of *E. necator* collected from naturally infected plants grown in an experimental field located in Miraflores, Curacaví, Chile (33°24′01.0″S 71°03′17.6″W) and maintained under *in vitro* conditions on grapevine leaves as described by Péros et al. (2005). Clonally propagated potted plants grown in greenhouse were used as source of young leaves. Leaves were inoculated with sporulating colonies of *E. necator* by gently tapping infected tissues above the adaxial leaf surface; petri dishes were sealed with Parafilm^®^ and placed in a growth chamber at 24 ± 2°C and 16/8 h photoperiod. Fungal cultures were renewed by re-inoculating fresh grapevine leaves every 2–4 weeks.

In order to genetically identify the fungal material used in this study, fungal samples were propagated as monosporangial cultures as described by Délye et al. (1997) and subcultured three times to ensure genetic uniformity of the fungal isolate. Fungal DNA extraction of three monosporangial cultures was performed as described by Montarry et al. (2009) and molecular characterization was done through a single nucleotide polymorphism (SNP) of the β-tubulin gene (GeneBank ID: AY074934) to distinguish genetically different groups A and B using cleaved amplified polymorphic sequence method, as described by Amrani and Corio-Costet (2006) and Montarry et al. (2009).

### Phenotypic and Genotypic Evaluation of Grapevine Segregating Plants

To evaluate the resistance trait, segregating plants were phenotyped in a greenhouse after infection with *E. necator*. Then, naturally infected plants showing white-grayish powder on the adaxial surface of the leaves were identified as susceptible. Later, this result was confirmed by *in vitro* inoculation with the fungus and phenotypic evaluation.

Subsequently, genomic DNA extraction of the segregating resistant plants was performed using the FavorPrep^TM^ Plant Genomic DNA Extraction Mini Kit (Favorgen Biotech Co., Taiwan). Positive controls to *RUN1* (02-2/81), *REN1* (91-4/27), and *RUN1REN1* (P09-105/34) were included.

Phenotypically resistant plants were screened using two simple sequence repeat (SSR) markers for each resistance locus. To identify *RUN1* genotypes, plants were screened with VMC4f3.1 and VMC8g9 markers (Barker et al., 2005). *REN1* genotypes were identified using Sc47_20 and UDV020 markers (Hoffmann et al., 2008; Coleman et al., 2009). Susceptible *V. vinifera* ‘Sauvignon Blanc’ and ‘Melissa’ were included as *RUN1* and *REN1* negative controls, respectively.

Additionally, *RUN1* genotypes were screened using a set of primers designed to specifically amplify a 190 bp fragment of the *MrRUN1* gene (GenBank ID: JQ904636): RUN1MG: F5′-ATAAAGCTCTTCGTATAAAT-3′ and R5′-CGATATGTGCTGACCCACA-3′. Susceptible *V. vinifera* ′Red Globe′ was included as *MrRUN1* negative control.

### Analysis of Fungal Proliferation and Plant Defense Response

To study fungal proliferation and plant defense response, *RUN1*, *REN1*, *RUN1REN1* genotypes and susceptible plants were directly inoculated with *E. necator* by gentle contact of infected tissue with the adaxial surface of the third to fifth fully expanded leaf from the apex and maintained in an infection greenhouse. Leaves were then harvested at 24, 48, and 96 hpi and analyzed as described below. All the assays included 106/7CS-40, ‘Thompson Seedless’ and ‘Crimson Seedless’ as susceptible genotypes.

#### Histological Assays

Fungal proliferation was studied by trypan blue staining as described by Vogel and Somerville (2000). For this, 10 mm diameter leaf disks were cut and washed in 96% ethanol. Later, disks from which chlorophyll was removed were stained with a 60°C preheated solution of 250 μg/mL trypan blue, water, 85% glycerol and lactic acid (1:1:1) for 30 min, then rinsed with the same solution lacking trypan blue overnight and mounted in slides for bright-field microscopy visualization of fungal structures. At least 100 stained conidia per biological replicate for each genotype were analyzed for fungal development. Infecting conidia were classified as ungerminated if no other structure developed from it, and germinated if appressoria and/or secondary hyphae developed. Germinated conidia were considered to achieve a successful penetration when secondary hypha development was observed. Conidia were scored in different inoculated leaf samples per each infection time.

Reactive oxygen species generation was evaluated in leaf disks infiltrated with 3,3′-diaminobenzidine (DAB)-HCl, pH: 3.8, for 7 min, incubated at room temperature and light conditions for 4 h (Thordal-Christensen et al., 1997), washed in 80% ethanol at 60°C for 10 min and ethanol 96% for 5 min.

Cell death was evaluated by staining with 250 μg/mL trypan blue in lactophenol (water, basic phenol, lactic acid and glycerol in a 1:1:1:1 ratio), incubating in boiling water for 3 min and rinsing in lactophenol at room temperature for 16 h, as described by Armijo et al. (2013). Samples were mounted on slides and examined under bright-field microscopy.

Callose deposits were studied by boiling leaf disks in lactophenol for 2 min, rinsing twice in 50% ethanol, twice in water, then staining with 0.01% aniline blue in 0.15 M K_2_HPO_4_ for 1 h and washing twice with water, according to Dietrich et al. (1994), and visualized under epifluorescence microscopy using a 365 nm UV filter.

All the microscopical observations, bright-field and epifluorescence, were made in a Nikon Eclipse 80i microscope (Nikon Instruments Inc., Tokyo, Japan).

#### Physiological Assays

Plant hormones SA and jasmonoyl-isoleucine (JA-Ile) were extracted and analyzed as described in Pan et al. (2010). Briefly, grapevine leaves (200 mg) were frozen in liquid nitrogen and homogenized in a mortar with 30% (v/v) isopropanol-15 mM HCl. The mixture was shaken at 4°C and centrifuged at 14000 rpm for recovering alcoholic solution containing hormones. The alcoholic supernatant was concentrated and plant hormones were detected and quantified using an HPLC–ESI–MS/MS system (Agilent 1200 series, MS/MS 5420). Samples were collected at 0, 48, and 96 hpi, using three biological replicates per genotype.

#### Molecular Assays

Total RNA was isolated from *RUN1*, *REN1*, *RUN1REN1* and susceptible inoculated leaf samples at 0, 24, 48, and 96 hpi, using three biological replicates per genotype. CTAB-spermidine buffer was used according to the procedure of Reid et al. (2006). For cDNA synthesis, samples containing 1 μg of total RNA were treated with *RQ1 RNase-free DNase* (Promega Corp., Madison, WI, USA) and reverse transcribed using random primers and SuperScript II RT (Invitrogen^TM^ Co., Carlsbad, CA, USA), following the manufacturer’s instructions. qPCR analyses were performed using the SensiMix^TM^ SYBR Hi-ROX Kit (Bioline, London, UK) and the Mx3000P qPCR system (Stratagene, Agilent Technologies Inc., Santa Clara, CA, USA) according to the manufacturer’s protocol.

Primers used in RT-qPCR corresponded to *VvCalS1* (ID: VIT_13s0156g00210): F5′-CAAAGGTGGAAAATCAAAGC-3′ and R5′-AGGCAGACGAAAGAAATCAG-3′; *VvWRKY27* (VIT_18s0001g10030): F5′-GACTTTGTGCTTGGGTGTCT-3′and R5′-TGGGGGTTTTCTACATTTCT-3′; *VvPR5* (VIT_02s0025g04330): F5′-CTCAGGATGACAAAACCAG-3′ and R5′-CACCAAGAAAGTGAAGGAAA-3′; *VvSTS36* (VIT_18s0001g07860): F5′-CTTGAAGGGGGAAAATGCT-3′ and R5′-TTACTGCATTGAAGGGTAAACC-3′; *MrRUN1*: F5′-CCTGAAGCGGAAATTCTCAG-3′ and R5′-TGCATGGAAATCACAAGCATCT-3′; *VvPEN1* (VIT08s0032g01150): F5′-CTTCGCAAGAAGCTCAGGGA-3′ and R5′-TGCTCTTGGATCGCCTTCTG-3′; and *Vv60SRP* (VIT_05s0077g02060): F5′-ATCTACCTCAAGCTCCTAGTC-3′ and R5′-CAATCTTGTCCTCCTTTCCT-3′. Expression levels of all the evaluated genes were calculated from three biological replicates, relative to *Vv60SRP* housekeeping gene and normalized by T0, using the ΔΔC_T_ method (Livak and Schmittgen, 2001).

### Statistical Analysis

All data presented in this study correspond to the mean ± SE, considering *n* = 3 for each sample. Results were subjected to one-way analysis of variance (ANOVA) and Bonferroni’s multiple comparison test (*P* ≤ 0.05).

## Results

### *E. necator* Isolates Belong to a Main Genetic Group

**FIGURE 1:**
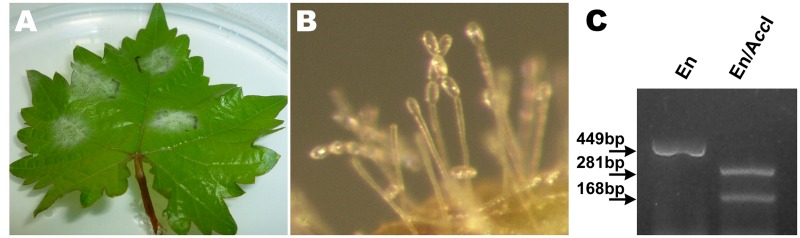
**Molecular characterization of *Erysiphe necator* in monosporangial cultures through single nucleotide polymorphism of β-tubulin gene.** **(A)** *In vitro* establishment of *E. necator* isolates in *Vitis vinifera* leaves in a dual culture technique. **(B)** Microscopic visualization of chains of *E. necator* conidia suitable for monosporangial culture. **(C)** Identification of the *E. necator* genetic group B using cleaved amplified polymorphic sequence method. En: isolate of *E. necator*; *Acc*I: restriction endonuclease; digestion by *Acc*I into two DNA fragments indicates the presence of group B isolate.

Single nucleotide polymorphism of *E. necator* β-tubulin to discriminate genetically different groups A and B showed that all the isolates analyzed carry the restriction site recognized by A*cc*I present only in the genetic group B. Thus, all the fungal samples propagated and used as inoculum in all the assays have B genotype (**Figure 1**). As we expected, it correlates with previous studies by Araya et al. (2014) that reported high frequency of group B isolates (98%) in Chilean vineyards, validating our inoculum source for further analysis.

### Complete Resistance to *E. necator* Is Accomplished by the Presence of At least One Resistance Locus

**FIGURE 2:**
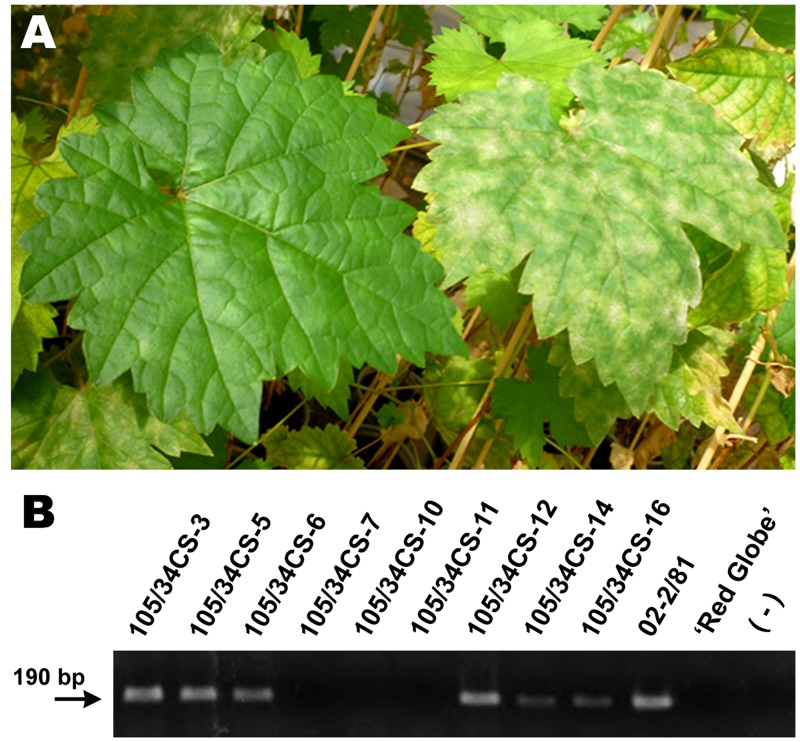
**Phenotypic and genotypic analyses of segregating grapevine plants.** **(A)** Phenotyping of plants resistant to infection. Right: powdery mildew development on a susceptible genotype; Left: leaf of resistant segregant carrying *RUN1* and/or *REN1* loci. **(B)** Genotyping of resistant plants carrying *MrRUN1*, by using specific primers. 105/34CS: grapevine segregating plants resistant to *E. necator*; 02-2/81: *MrRUN1* positive control; ‘Red Globe’: *MrRUN1* negative control; (-): DNA negative control; arrow shows a 190 bp DNA fragment expected to amplify in *MrRUN1* positive plants.

Naturally infected resistant segregants and susceptible control plants showed two differential phenotypes that allowed to unequivocally distinguish which plants were resistant or susceptible to *E. necator* infection. All the resistant segregants exhibited complete resistance with no signs or symptoms of grape powdery mildew (**Figure 2A**). Dense mycelium and conidiophore development was only observed in susceptible plants.

In order to genetically identify whether resistant segregants correspond to *RUN1*, *REN1* or *RUN1REN1* genotypes, we analyzed them with SSR markers that co-segregate with *RUN1* and *REN1*. Three individuals per each genotype were obtained (**Table 1**). The results obtained with VMC4f3.1 and VMC8G9 were confirmed with the set of primers that specifically target *MrRUN1* gene (**Figure 2B**). Thus, we confirmed the reliability of our designed primers to easily identify the presence of *MrRUN1* and also corroborated the identity of the genotypes carrying the *RUN1* locus.

**Table 1:** Marker assisted selection of grapevine segregants resistant to *Erysiphe necator*, carrying *RUN1* and/or *REN1* loci.

	*RUN1* SSR markers		REN1 SSR markers		
Segregant					Genotype
	VMC4f3.1	VMC8g9	Sc47_20	UDV020	
105/34CS-3	+	+	+	+	*RUN1REN1*
105/34CS-5	+	+	-	-	*RUN1*
105/34CS-6	+	+	-	-	*RUN1*
105/34CS-7	-	-	+	+	*REN1*
105/34CS-10	-	-	+	+	*REN1*
105/34CS-11	-	-	+	+	*REN1*
105/34CS-12	+	+	+	+	*RUN1REN1*
105/34CS-14	+	+	+	+	*RUN1REN1*
105/34CS-16	+	+	-	-	*RUN1*
02-2/81^a^	+	+	-	-	*RUN1*
91-4/27^b^	-	-	+	+	*REN1*
P09-105/34^c^	+	+	+	+	*RUN1REN1*
Sauvignon Blanc^d^	-	-	-	-	Susceptible
Melissa^e^	-	-	-	-	Susceptible


*^a^Included as *RUN1* positive control.*

*^b^Included as *REN1* positive control.*

*^c^Included as *RUN1REN1* positive control.*

*^d^Included as *RUN1* negative control.*

*^e^Included as *REN1* negative control.*

### Pyramiding of *RUN1* and *REN* Loci Displayed a Failure in Penetration and Subsequent Invasion of the Grapevine Tissues

**FIGURE 3:**
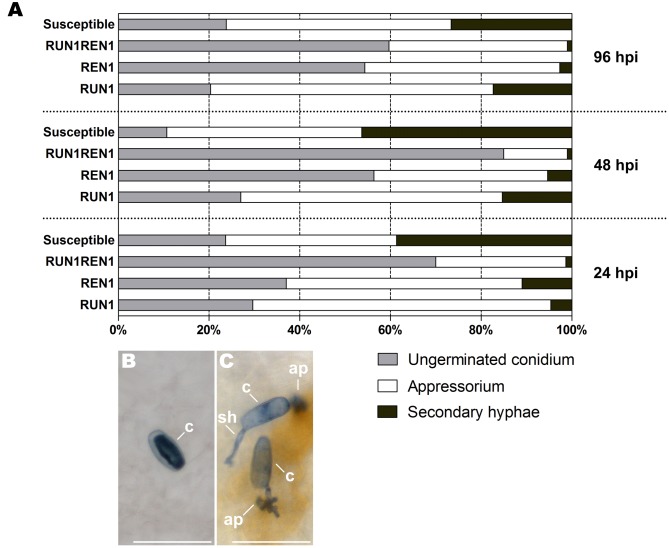
**Development of *E. necator* in leaves of resistant grapevine genotypes carrying *RUN1* and/or *REN1* loci.** **(A)** Frequency of ungerminated conidia, penetration attempts leading to appressorium formation but unsuccessful penetration, and penetration attempts leading to successful penetration with secondary hypha development at 24, 48, and 96 hours post-inoculation (hpi). Each data point was calculated from three biological replicates by scoring a minimum of 100 inoculated conidia per leaf disk. Conidia were scored in different inoculated leaf samples per each infection time. **(B)** Ungerminated conidia; **(C)** germinated conidia exhibiting successful (top) and restricted (bottom) penetration. c, conidium; ap, appressorium; sh, secondary hypha; scale bar, 50 μm.

In the course of the whole experiment, *E. necator* conidia showed germination rates between 76 and 89% in susceptible genotypes and also 34–51% of them successfully penetrated plant cells and developed secondary hypha, thus indicating an establishment and proliferation of the pathogen on grapevine leaf tissues. On the contrary, the three resistant genotypes (*RUN1*, *REN1*, and *RUN1REN1*) showed a restricted development and proliferation of *E. necator* throughout the infection kinetics, compared to susceptible plants, displaying around 83 and 99% of the total conidia lacking secondary hypha development (**Figure 3**). In terms of conidia development, *RUN1REN1* genotype was the most restrictive where only 1–1.3% of the total conidia could penetrate plant cells and form secondary hypha. Interestingly, increased rate of ungerminated conidia occurred in *RUN1REN1* genotypes from early infection times, representing among 60 and 85% compared to *RUN1* and *REN1* genotypes. The remaining conidia displayed only 2–5% of successful penetration. In the case of *RUN1* and *REN1* genotypes, successful penetration rates of the germinated conidia were higher than those from the pyramided genotype with values up to 21 and 16%, respectively (**Figure 3A**).

Pyramiding of *RUN1* and *REN* loci displayed an enhanced resistance phenotype leading to a failure in penetration and subsequent invasion of the grapevine tissues, preventing the future fungus dispersion. This negative effect on conidia germination and penetration was even greater than in genotypes carrying single resistance locus from early infection stages.

### The Presence of *RUN1* and/or *REN1* Locus efficiently Restricted the Fungal Development through ROS Generation, Callose Deposition, and PCD

In order to elucidate the defense response given by the pyramiding of both resistance loci, we visualized the plant defense response against *E. necator* in *RUN1* and *REN1*, single and double loci segregating plants by histochemical analysis.

**FIGURE 4:**
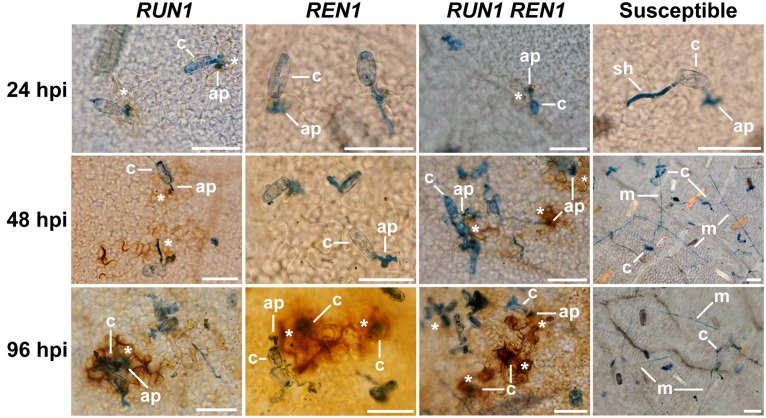
**Reactive oxygen species (ROS) generation at the *E. necator* infection site in segregating grapevine plants carrying *RUN1* and/or *REN1* loci.** Samples were collected at 24, 48, and 96 hpi; c, conidium; ap, appressorium; sh, secondary hypha; m, mycelium; asterisks indicate brown diaminobenzidine-stained ROS in response to infection; scale bar, 50 μm.

First, we studied the temporality and occurrence of the oxidative burst by microscopic observations of DAB-stained ROS generated at the infection site (**Figure 4**). Susceptible plants exhibited a normal establishment and development of the fungus over grapevine leaves surface in all the infection times, showing successful penetration and secondary hypha development with profuse mycelia and absence of ROS generation. On the contrary, resistant plants carrying *RUN1* locus, single or pyramided, showed ROS generation from 24 hpi at the infection sites, whereas in REN1 genotypes ROS staining could be clearly observed only at 96 hpi. At 48 hpi, cells attempted to be penetrated were notoriously brown-stained in *RUN1* and *RUN1REN1* genotypes. At the last time, infected cells stained intensely brown. The presence of *RUN1* and/or *REN1* locus efficiently restricted the development of mycelia. Although the defensive ROS generation occurred in all the resistant genotypes, *REN1* genotypes displayed a delayed defense response compared to plants carrying *RUN1* (**Figure 4**).

**FIGURE 5:**
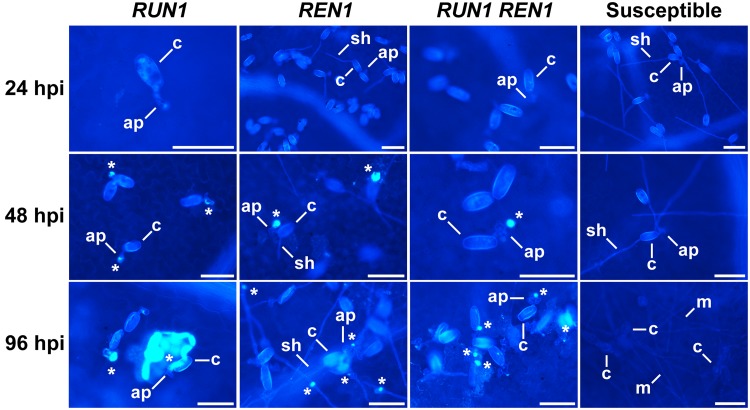
**Accumulation of callose deposits at the *E. necator* infection site in segregating grapevine plants carrying *RUN1* and/or *REN1* loci.** Samples were collected at 24, 48, and 96 hpi; c, conidium; ap, appressorium; sh, secondary hypha; m, mycelium; asterisks indicate bright-blue fluorescent callose accumulation in response to infection; scale bar, 50 μm.

We also studied the defense response against powdery mildew mediated by the accumulation of callose deposits at the infection site. In susceptible genotypes conidia were able to germinate, penetrate the plant cells and develop secondary hypha from early infection stages until 96 hpi exhibiting dense mycelia and, as expected, absence of callose deposits. Conversely, all the resistant genotypes accumulated callose deposits, as shown by the bright-blue spots at the infection site from 48 hpi (**Figure 5**). At 96 hpi, almost every single penetration site showed callose deposits in *RUN1* and *RUN1REN1* genotypes while in *REN1* genotypes we observed a higher number of penetration sites that lacked of bright-blue fluorescence. The differential response of plants carrying only *REN1* locus could also be correlated with a more successful fungal development compared to plants carrying *RUN1* alone or together with *REN1*, although less extensively than in susceptible genotypes (**Figure 5**).

**FIGURE 6:**
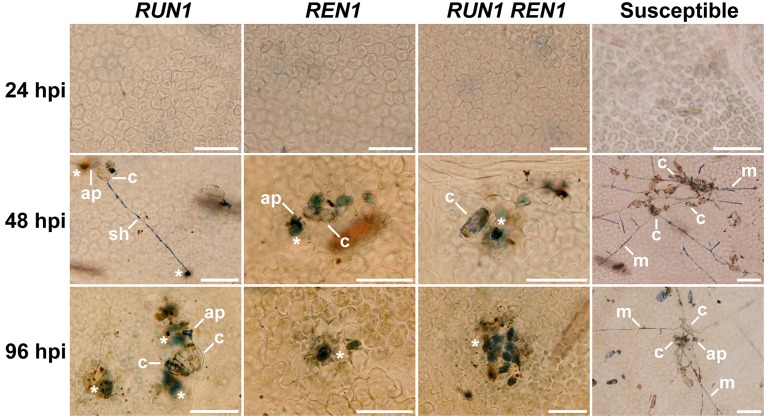
**Induction of programmed cell death (PCD) at the *E. necator* infection site in segregating grapevine plants carrying *RUN1* and/or *REN1* loci.** Samples were collected at 24, 48, and 96 hpi; c, conidium; ap, appressorium; sh, secondary hypha; m, mycelium; asterisks indicate trypan blue-stained cells undergoing PCD in response to infection; scale bar, 50 μm.

Finally, we examined the occurrence of PCD in the infection site as a final response triggered to stop fungal development and avoid the spread of *E. necator*. At 24 hpi, susceptible and resistant genotypes showed absence of blue-stained cells undergoing PCD. As expected, in susceptible genotypes no visible PCD was detected through the entire infection kinetics. In resistant genotypes, it was possible to observe blue-stained cells being penetrated by appressoria from 48 hpi. At 96 hpi, this defense response was markedly stronger in genotypes carrying *RUN1* alone than *REN1*, but it was even stronger in genotypes carrying both pyramided loci (**Figure 6**). Even when this staining procedure was optimized for plant cell death, some fungal structures were poorly stained and we could rarely observe secondary hypha development in all the resistant genotypes studied.

### Differential Expression Patterns of Defense-Related Genes in Resistant Genotypes

**FIGURE 7:**
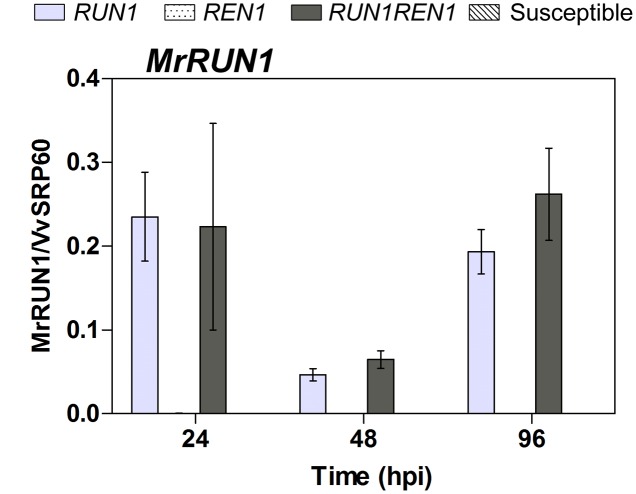
**RT-qPCR analysis of *MrRUN1* expression in response to *E. necator*.** Samples were collected at 24, 48, and 96 hpi. Expression data were related to *Vv60SRP* housekeeping gene. Data points represent means ± SEM considering three biological replicates.

Since *MrRUN1*, a TIR-NB-LRR gene that confers resistance to *E. necator* was identified (Feechan et al., 2013a), we analyzed its relative expression levels in all the plants studied. As expected, we observed *MrRUN1* induction only in *RUN1* and *RUN1REN1* genotypes. Expression patterns confirmed the activation and functionality of this gene in all the infected resistant segregants carrying *RUN1* in our studies, showing no difference mediated by pyramiding both resistance loci (**Figure 7**).

**FIGURE 8:**
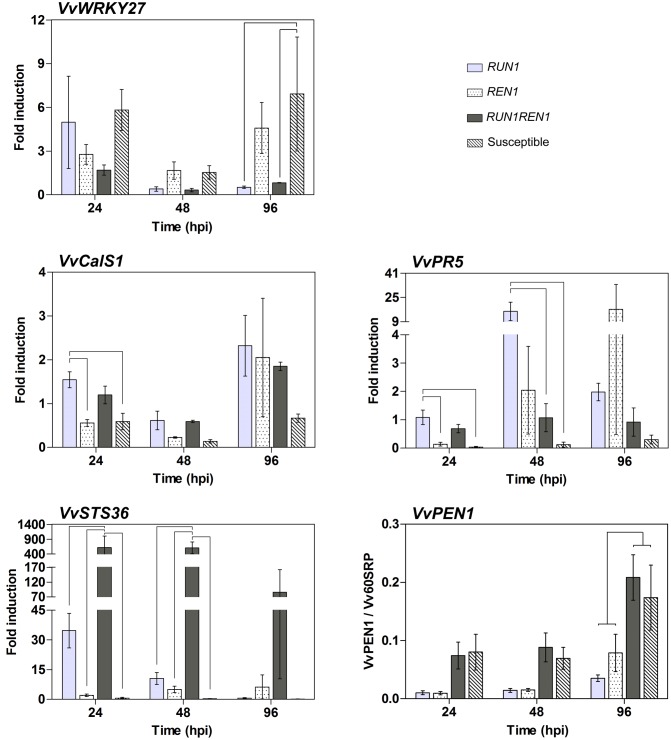
**RT-qPCR analysis of defense-related gene expression in response to *E. necator*.** Samples were collected at 24, 48, and 96 hpi. Expression data were related to *Vv60SRP* housekeeping gene and/or normalized to uninfected controls (0 hpi). Data points represent means ± SEM considering three biological replicates. Linking lines shows statistically significant difference between genotypes as determined by Bonferroni’s multiple comparison test (*P* ≤ 0.05).

To further understand the grapevine defense response against *E. necator* associated with *RUN1* and/or *REN1* loci, we studied the expression patterns of genes related to immune response in plants. The analysis of *VvWRKY27*, a transcription factor involved in defense response against *E. necator* (Guo et al., 2014), showed the highest expression levels in susceptible genotypes, showing a significantly higher induction compared to *RUN1* and *RUN1REN1* plants at 96 hpi (**Figure 8**). Among the resistant genotypes, those carrying only *RUN1* reached an induction of 5-fold at 24 hpi, almost 10-fold higher than at later times of infection. Interestingly, in plants carrying both resistance loci, *VvWRKY27* induction was lower than single locus genotypes, significantly decreasing from 1.7-fold to 0.3-fold between 24 and 48 hpi, respectively.

We also studied *VvCalS1*, involved in callose biosynthesis and thus related to the accumulation of callose deposits (or papilla) at the infection site as a defense response against penetration attempts and establishment of phytopathogenic microorganisms in plant tissues (Yu et al., 2016). The lowest induction of this gene was observed in the susceptible genotypes during the complete infection kinetics with values between 0.1-fold and 0.7-fold (**Figure 8**). At 24 hpi, *VvCalS1* 1.5-fold induction in response to *E. necator* infection was significantly higher in plants carrying only *RUN1* than *REN1* and susceptible genotypes. All the resistant genotypes showed a tendency of induced expression of this gene at 24 hpi followed by a decrease at 48 hpi and then a marked increase at 96 hpi. However, the differences remained below the significance threshold, except for the *RUN1REN1* genotypes, where the induction significantly decreased from 1.2-fold to 0.6-fold between 24 and 48 hpi, followed by a significant increase to 1.9-fold at 96 hpi.

*VvPR5*, a thaumatin-like protein involved in plant defense (Dhekney et al., 2011), displayed the lowest induction in susceptible genotypes throughout the infection times analyzed, varying between 0.03-fold at 24 hpi and 0.3-fold at 96 hpi (**Figure 8**). Genotypes with pyramided *RUN1* and *REN1* also responded to the infection with constant low induction of *VvPR5* through all phases of infection compared to genotypes carrying only *RUN1* or *REN1* locus. *RUN1REN1* plants showed between 0.7-fold and 1.1-fold induction but differences with the other resistant genotypes remained below the level of significance, except at 48 hpi where induction in *RUN1REN1* and susceptible genotypes resulted in significantly lower induction than *RUN1* genotype (**Figure 8**). Regarding genotypes carrying *RUN1* or *REN1* alone, *VvPR5* responded with a tendency to strong induction in later infection times, with 16-fold at 48 hpi and 17-fold at 96 hpi for *RUN1* and *REN1* genotypes, respectively.

*VvSTS36*, coding for an enzyme involved in stilbene synthesis that accumulates in response to biotic stress (Vannozzi et al., 2012), was poorly induced in genotypes lacking resistance loci, between 0.1-fold and 0.6-fold through all infection times (**Figure 8**). However, *RUN1* genotypes showed a 35-fold induction at 24 hpi, significantly decreasing to 11-fold and 0.6-fold induction at 48 and 96 hpi, respectively. In the case of *REN1* genotypes, *VvSTS36* was induced in a range from 2-fold to 6.3-fold throughout the infection phases. On the contrary, genotypes carrying both pyramided resistance loci reached a higher *VvSTS36* induction in all post-inoculation times, reaching up to 620-fold and 606-fold induction at 24 and 48 hpi, respectively, significantly higher than single locus and susceptible genotypes (**Figure 8**).

*VvPEN1*, gene related to penetration resistance against powdery mildew (Feechan et al., 2013b), showed a constantly higher induction in *RUN1REN1* and susceptible genotypes than plants carrying single R locus during all the infection phases, reaching statistically significance at 96 hpi (**Figure 8**).

### Differential Levels of Plant Hormones in Response to Powdery Mildew in Resistant Genotypes

**FIGURE 9:**
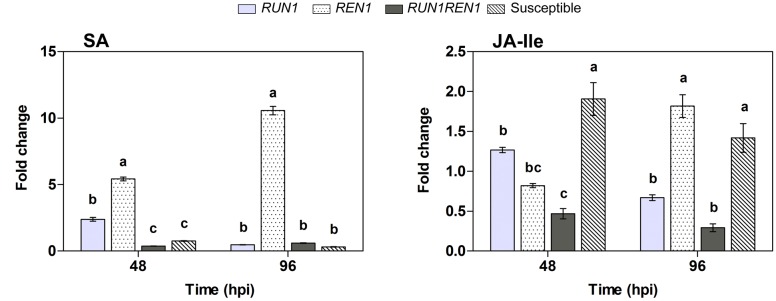
**Analysis of salicylic acid (SA) and jasmonoyl-isoleucine (JA-Ile) levels in response to *E. necator*.** Samples were collected at 0, 48, and 96 hpi; data were normalized to uninfected controls (0 hpi). Data points represent means ± SEM considering three biological replicates. Different letters indicate statistically significant difference between genotypes as determined by Bonferroni’s multiple comparison test (*P* ≤ 0.05).

Plants carrying *RUN1* and *REN1* pyramided loci showed lower SA and JA-Ile levels, hormones involved in defense response (Casagrande et al., 2011; Qiu et al., 2015), compared with the other genotypes (**Figure 9**). In these plants SA levels showed a 0.6-fold induction at 96 hpi. On the contrary, *REN1* genotypes showed the highest levels at both infection times, up to 10.6-fold change at 96 hpi, in response to *E. necator*.

Jasmonoyl-isoleucine levels changed only up to 0.5-fold at 48 hpi in *RUN1REN* genotypes, significantly lower than 1.3-fold change in *RUN1* plants. At 96 hpi, JA-Ile level in *REN1* genotype increased 2-fold change and was significantly higher than level in the *RUN1REN1* pyramided genotype (**Figure 9**).

## Discussion

Resistance to pathogenic microorganisms is a common and important trait to be incorporated in new plant cultivars. Many sources of resistance to grapevine powdery mildew have been identified, including some North American, Chinese species, and even some Asian *V. vinifera* cultivars, showing different levels of resistance but lacking commercial qualities (Barker et al., 2005; Welter et al., 2007; Ramming et al., 2011; Blanc et al., 2012; Feechan et al., 2015; Pap et al., 2016). In this work, we used segregating plants from *V. vinifera* ‘Dzhandzhal Kara’ × *V. vinifera* ‘Laszta’ and the fifth pseudo-backcross of *M. rotundifolia* × *V. vinifera* as two genetically different sources of resistance against the biotrophic fungus *E. necator* carrying *REN1* and *RUN1* locus, respectively, and pyramided them in single grapevine plants until the seventh pseudo-backcross with *V. vinifera* ‘Crimson Seedless’ (**Supplementary Figure S1**).

In previous works, hybrid grape populations carrying the same pyramided loci were developed and analyzed with the purpose of detecting them through marker assisted selection (Katula-Debreceni et al., 2010; Li et al., 2013), but the progenies used corresponded only to the fifth and sixth backcross and therefore the genetic component has a lower percentage of *V. vinifera* in comparison with our developed progeny. Furthermore, plant defense mechanism triggered by *RUN1* and *REN1* was not studied.

*Muscadinia rotundifolia* has been described to show complete resistance, allowing low levels of penetration but rapidly inducing PCD near 75% of the infected cells to successfully halt secondary hypha elongation, avoiding sporulation (Dry et al., 2010; Feechan et al., 2011). Similar results were observed in transgenic plants overexpressing *MrRUN1*, the responsible gene for this resistance (Feechan et al., 2013a). Our results also showed that *RUN1* genotypes restrict the development and proliferation of infected conidia, with very low rates of successful penetration into the inoculated cells compared to susceptible genotypes, involving a very effective reaction at the site of infection with rapid ROS generation, callose accumulation and induction of PCD from the first 24–48 hpi.

In the case of ‘Kishmish Vatkana,’ one of the sources of *REN1* locus, PCD induction in penetrated cells and restriction of hyphal development has been described, but with a slower intensity than in the case of *RUN1*-mediated resistance (Hoffmann et al., 2008; Qiu et al., 2015). Our results showed that *REN1* genotypes highly decreased the rate of secondary hypha development compared to susceptible plants, but halting of hyphal growth and temporality of the response was delayed compared to *RUN1* plants. Epidermal cells responded to infection with ROS generation at 96 hpi. At earlier times, only a very scarce and slight soft brownish staining appeared under appressoria. Epidermal cells under penetration attempts also responded by accumulating callose deposits and inducing PCD to halt fungal growth from 48 hpi but callose deposits were apparently less frequent and PCD staining weaker than those observed in *RUN1* genotypes at 96 hpi. These results indicate that *REN1* genotypes display a delayed defense response compared to *RUN1* genotypes, but equally strong to avoid fungal colonization and proliferation.

Our main results concern the defense mechanism displayed in the presence of both *RUN1* and *REN1* loci in one grapevine genotype. Epidermal cells responded against *E. necator* infection by an effective and strong defense response from early infection times. Results showed strong ROS generation at the infection site from 24 hpi, followed by callose deposit accumulation in the most of penetration attempts and induction of PCD from 48 hpi. In terms of fungal establishment, results showed that the defense mechanism triggered by the pyramiding of *RUN1* and *REN1* leads to extremely low rates of successful penetration and subsequent hypha elongation, even lower than in the case of *RUN1* or *REN1* independently. Besides, we consistently observed a strong detrimental effect on conidia germination only in *RUN1REN1* genotypes. The latter could be correlated with localized early responses at the conidial contact site, including plant response gene transcription, originated by elicitors from the surfaces of the conidia (Zeyen et al., 2002). Thus, our results reveal an additional enhanced resistance to *E. necator* provided by the *RUN1* and *REN1* pyramiding. Additional resistance has been previously reported in grapevine genotypes in which both *RUN1* and *REN2* loci are combined (Feechan et al., 2015).

Besides the cellular response against *E. necator*, we studied the expression of some genes related to plant defense response to elucidate molecular aspects involved in *RUN1* and/or *REN1*-mediated resistance mechanism. *WRKY* transcription factors play an important role in signal transduction systems and act as global regulators of host responses in pathogenesis, leading to an activation of plant defense reaction against various pathogens (Vidhyasekaran, 2007; Pandey and Somssich, 2009; Merz et al., 2015). In grapevine, *VvWRKY27* activation has been associated with a positive effect on powdery mildew resistance (Guo et al., 2014). Our results showed a high induction of *VvWRKY27* in *E. necator-V. vinifera* compatible interaction. In plants carrying *RUN1* alone or combined with *REN1*, high expression of *VvWRKY27* only occurred at the earliest infection time. It suggests an important role of this gene on the induction of basal defense response against *E. necator*, with no or little involvement on the resistance mechanism mediated by *RUN1REN1* genes. Fung et al. (2008) also detected increased levels of transcripts encoding *WRKY* transcription factors in susceptible *V. vinifera* but not in resistant *V. aestivalis*, to a defense-oriented transcriptional change in *V. vinifera*. Other *WRKY* genes, as *VvWRKY33, VpWRKY1*, and *VpWRKY2* have been related to grapevine resistance against pathogens (Li et al., 2010; Merz et al., 2015; Qiu et al., 2015).

*CalS*, or callose synthase, corresponds to a class of enzymes involved in callose biosynthesis in plants (Chen and Kim, 2009), a minor component of healthy plant tissue which rapidly accumulates around the infection site in order to reinforce cell walls and prevent fungal invasion (Vidhyasekaran, 2007; Hückelhoven, 2014; Kuhn et al., 2016). *CalS* activity has been correlated with enlarged callose deposits (Ellinger et al., 2013). We observed low induction levels of *VvCalS1* in susceptible genotypes compared to resistant genotypes, and *RUN1* plants showed significantly higher induction of this gene than *REN1* plants. The latter is well correlated with the delayed defense response mediated by *REN1* locus, which allows advanced secondary hypha development, observed in the aniline blue staining analysis. Our results also reveal an early *VvCalS1* induction in resistant genotypes followed by a higher induction at 96 hpi, suggesting a role in both basal defense and ETI-response against *E. necator*, in accordance with other studies where papilla formation has been seen in incompatible and compatible interactions (Vidhyasekaran, 2007). Among others, efficacy of papillae as a resistance mechanism would depend on callose accumulation, early initiation, size and frequency of papilla (Vidhyasekaran, 2007; Chowdhury et al., 2014; Hückelhoven, 2014). Yu et al. (2012) also described callose deposits in *M. rotundifolia* and *V. pseudoreticulata* from 24 to 192 hpi in response to *P. viticola*, but not in susceptible genotypes. *VvCalS1* and *VvCalS10* have been proposed to be involved in defense against grapevine downy mildew (Yu et al., 2016).

Pathogenesis-related proteins correspond to a family of proteins known to appear and accumulate during plant defense response (Vidhyasekaran, 2007). *PR5*, a thaumatin-like protein described to alter the permeability of fungal membranes, has been reported to play a role in grapevine-pathogen interaction (Monteiro et al., 2003; Vidhyasekaran, 2007; Chang et al., 2011; Dhekney et al., 2011; Gray et al., 2014; Weng et al., 2014). Our results showed very low induction levels of *VvPR5* in susceptible genotypes, low induction levels in *RUN1REN1* genotypes and, in general, higher induction in the remaining resistant genotypes carrying only *RUN1* or *REN1* loci. This suggest a minor input of *VvPR5* in the resistance mechanism mediated by both *RUN1* and *REN1* pyramided loci compared to the contribution of this gene in the single *RUN1* or *REN1* resistance defense response against *E. necator*. It may represent the existence of strong defense machinery in *RUN1REN1* genotypes, reducing the need for *VvPR5* expression. Monteiro et al. (2003) described the induced expression of a thaumatin-like protein in grapevine leaves and berries after *E. necator* infection. Previous studies have also shown strong *PR5* induction in the resistant *V. quinquangularis* ‘Shang-24’ in response to *E. necator* inoculation but not in susceptible plants (Gao et al., 2012). Fung et al. (2008) reported increasing induction levels of *PR2, PR3*, and *PR5* across powdery mildew infection in grapevine. Moreover, *VvPR5* is also induced in an incompatible interaction with other grapevine pathogens as *P. viticola* (Malacarne et al., 2011).

Another important gene involved in resistance to pathogens is *STS*, or stilbene synthase. It corresponds to the key enzyme involved in the biosynthesis of stilbene phytoalexins, i.e., resveratrol (Vidhyasekaran, 2007; Chong et al., 2009). Previous studies have demonstrated that grapevine stilbenes strongly accumulate in response to several biotic stresses, including powdery mildew infection (Fung et al., 2008; Schnee et al., 2008; Vannozzi et al., 2012). In our results, we observed minimal *VvSTS36* induction in susceptible genotypes throughout all phases of infection and low levels of induction in the single *RUN1* or *REN1* genotypes, except for *RUN1* plants where we observed an initial higher *VvSTS36* induction compared to latter infection times, in agreement with Alonso-Villaverde et al. (2011) who supposed a rapid induction of metabolic responses as the most important part of disease inhibition in *M. rotundifolia*, which accumulates large concentrations of stilbenes in response to downy mildew. In correlation with the pyramided *RUN1REN1* genotype, we observed a very strong induction of *VvSTS36* in response to *E. necator* infection in all post-inoculation times, suggesting that stilbenes could be an important component of the resistance mechanism mediated by *RUN1* and *REN1*. Besides, this consistently strong *VvSTS36* induction could be related to the observed very low conidium germination, as it has been described for other plant–fungus interactions (Vidhyasekaran, 2007). In accordance with our results, the synthesis induction and antimicrobial activity of plant stilbenes have been suggested to be a key part of defense responses and also an indicator of disease resistance and thus, a positive correlation exists between resveratrol production (and its derivatives) and resistance of *Vitis* spp. to biotrophic pathogens (Fung et al., 2008; Schnee et al., 2008; Chong et al., 2009; Jeandet et al., 2010; Malacarne et al., 2011; Huang et al., 2016; Jiao et al., 2016) and at least 20 *VvSTS* would be expressing in grapevine followed infection with downy mildew (Richter et al., 2006; Chong et al., 2009).

*VvPEN1* corresponds to a member of the SNARE (soluble *N*-ethylmaleimide-sensitive factor attachment protein receptor) family and has been related to the trafficking of secretory vesicles to the plasma membrane, transporting the necessary cargo for penetration resistance against powdery mildew (Qiu et al., 2015). *PEN1*- and *PEN2/PEN3*-like pathways are suggested as important components of pathogen-associated molecular patterns (PAMP)-triggered immunity (PTI) in grapevine. In previous studies, *VvPEN1* was used for functional complementation of the *Arabidopsis thaliana pen1* mutant and also it has been observed that *VvPEN1*-GFP fusion protein accumulates under the site of powdery mildew penetration (Bhat et al., 2005; Feechan et al., 2013b). We observed high induction levels of this gene in susceptible genotypes, possibly related to basal defense, and also in plants carrying both *RUN1* and *REN1* loci. These results indicate that *VvPEN1* would play an important role in the basal defense response triggered in *RUN1REN1* genotypes at the site of infection. Induction levels of both *VvPEN1* and *VvSTS36* suggests that early defense response would be a critical component in *RUN1REN1* genotypes, restricting conidia germination and also the activation of a PTI-like response affecting fungal establishment at the site of penetration.

Salicylic acid and JA-Ile, the bioactive form of jasmonate (JA), are two plant hormones implicated in plant defense response. In a previous work, transcriptomic analysis of *E. necator* infected leaves of the resistant *V. pseudoreticulata* identified a significant induction of genes belonging to defense, SA and JA responses, systemic acquired resistance (SAR), HR, plant–pathogen interaction, flavonoid biosynthesis and plant hormone signal transduction categories, suggesting that effective resistance responses of grapes to *E. necator* includes enhancement of JA and SA responses and accumulation of phytoalexins (Weng et al., 2014). We observed low levels of SA and JA-Ile in response to powdery mildew in plants carrying both *RUN1* and *REN1* pyramided loci, suggesting that the effective defense response displayed since early infection times would be enough to successfully defend themselves against *E. necator*. In agreement with this, *RUN1* and *REN1* genotypes showed differentially increased levels of JA-Ile and mainly SA, as plants infected by a biotrophic fungus.

An important issue in the development of new pathogen resistant cultivars is the emergence of new virulent isolates with the ability to overcome the R genes recognition (Peressotti et al., 2010; Cadle-Davidson et al., 2011). Hence, pyramiding two or more R genes from different *Vitis* species becomes a durable and secure strategy to adopt, even if any mutation or loss of an avirulence factor occurs, the pathogen will be still recognized by at least one R gene (Feechan et al., 2015; Armijo et al., 2016a; Pap et al., 2016).

Cadle-Davidson et al. (2011) reported signs of powdery mildew on *RUN1* positive plants in Geneva, New York, and the appearance of an *E. necator* virulent isolate Musc4 collected from *M. rotundifolia* (Feechan et al., 2015). Nevertheless, *RUN1* resistance is considered to still retain considerable commercial value if adequate breeding and management strategies to protect *RUN1* are adopted (Cadle-Davidson et al., 2011). *MrRUN1* is known to recognize and confer resistance to diverse isolates from Europe, Australia, North America (Feechan et al., 2013a), and here we report South American isolates as well. In addition to *RUN1*, the use of *REN1* has the advantage that it does not compromise the purity of *V. vinifera* genome when pyramided (Hoffmann et al., 2008). Recently, Rubio et al. (2015) have developed transgenic ‘Thompson Seedless’ lines with improved tolerance against *E. necator* using two endochitinase and one *N*-acetyl-β-d-hexosaminidase genes from *Trichoderma* spp., and also Feechan et al. (2013a) observed effective *MrRUN1-*mediated resistance to powdery mildew in transgenic vines expressing this TIR-NB-LRR gene, but it was lower than the defense response displayed in vines carrying the complete *RUN1* locus, probably due to minor contributions of other resistant gene analogs (RGAs) located in the *RUN1/RPV1* locus.

Our results suggest that *RUN1REN1* genotypes display a better early restriction of fungal establishment and colonization than one single locus plants by, for example, early stilbene synthase gene induction, increased penetration resistance and negatively affecting conidia germination. This could be related with activation of a PTI-like response affecting fungal establishment at the site of penetration, making unnecessary to trigger responses presented in plants carrying only *RUN1* or *REN1* loci in the same magnitude, to avoid *E. necator* proliferation. Different components of the entire *RUN1* and *REN1* loci in combination could be responsible of the positive effects leading to enhanced resistance, similarly to the better resistance observed in *RUN1*-positive versus only *MrRUN1*-positive transgenic vines by Feechan et al. (2013a) but at a better extent given by the addition of the *REN1* locus.

## Conclusion

Genotypes carrying both *RUN1REN1* pyramided loci display an effective and strong defense mechanism against powdery mildew, one of its most prevalent and detrimental fungal diseases of *V. vinifera*. Future transcriptomic and secondary metabolite analysis are needed to a better understanding of the incompatible interaction mediated by *RUN1* and *REN1* loci. Grapevine plants developed in this work have great potential as new table grape cultivar with durable complete resistance to *E. necator*. Hence, they can greatly reduce the chemical fungicide input, costs and environmental impact associated with grape production can efficiently be lowered. Besides, by co-segregation, they carry the *RPV1* locus for resistance to grape downy mildew; so they constitute valuable germplasm to be included in breeding programs for future pyramiding with other sources of resistance to powdery mildew and/or other grapevine diseases.

## Author Contributions

MA, RS, and GA conducted the experiments; ES contributed to replicate some experiments; CS contributed to the marker-assisted selection standardization and analysis; RC and GZ contributed to the physiological analysis. All the authors contributed to the analysis of the results and the writing of the manuscript.

## Conflict of Interest Statement

The authors declare that the research was conducted in the absence of any commercial or financial relationships that could be construed as a potential conflict of interest.

## Supplementary Material

**FIGURE S1 | Pseudo-backcrossing scheme of *Erysiphe necator* resistance sources with susceptible *Vitis vinifera* cultivars for *RUN1* and *REN1* pyramiding.** *Muscadinia rotundifolia*: source of *RUN1*; Dzhandzhal Kara: source of *REN1*; NC, VRH and 02-2/81: *RUN1*-positive genotypes; 91-4/27: *REN1*-positive genotype; P09-105/34: *RUN1REN1*-positive genotype; 105/34CS: segregating progeny carrying *RUN1* and/or *REN1*; BCn: pseudo-backcrossing that originated the individual (Hoffmann, personal communication).
