# Supplementary figures and images for: RUN1 and REN1 Pyramiding in Grapevine (Vitis vinifera cv. Crimson Seedless) Displays an Improved Defense Response Leading to Enhanced Resistance to Powdery Mildew (Erysiphe necator)
Source: Front Plant Sci. 2017 May 12;8:758. doi: 10.3389/fpls.2017.00758 (PMC5427124; doi:10.3389/fpls.2017.00758)

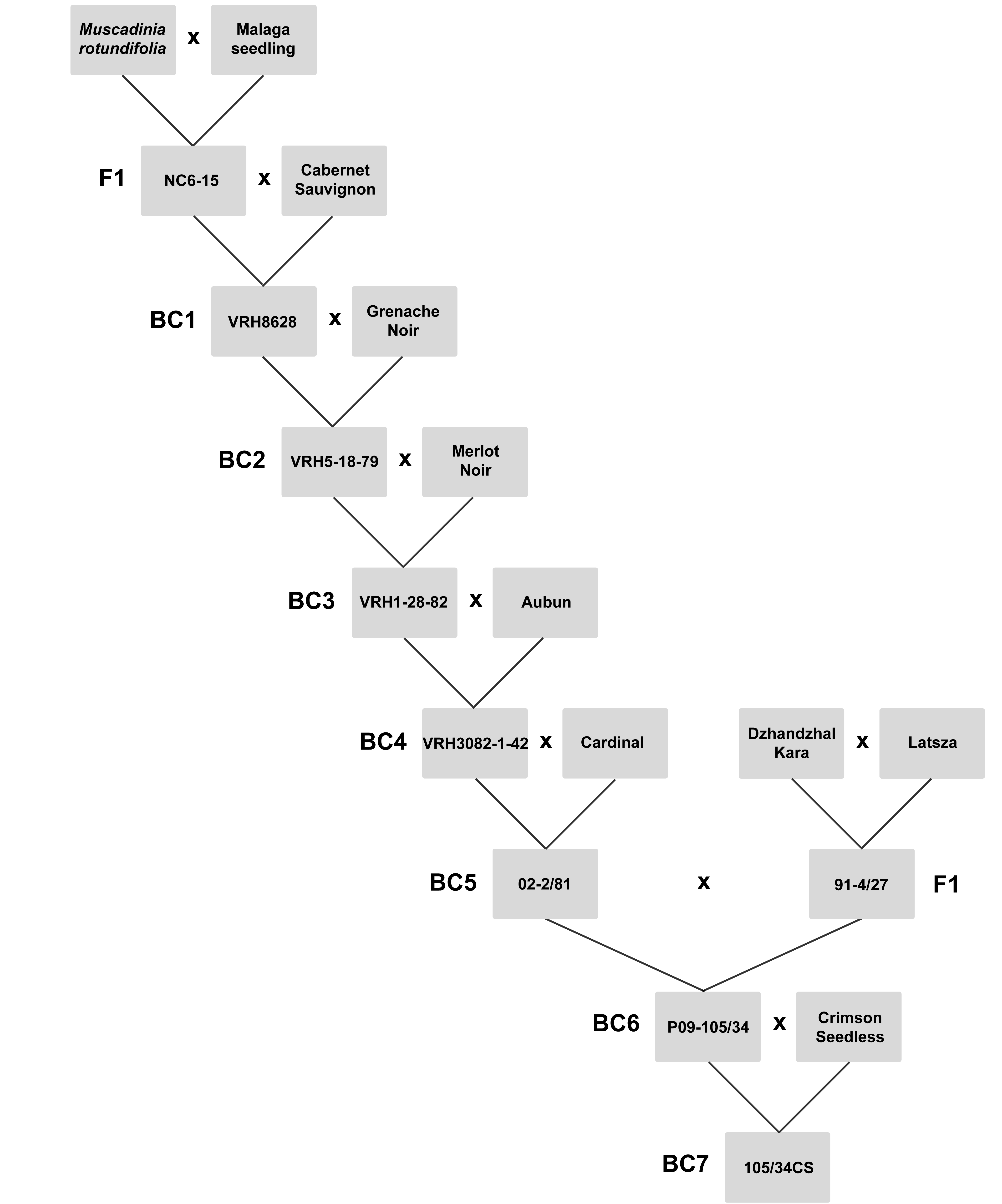

Supplement: Supplementary file 1 [file Image_1.TIF]
